# An explainable CT-based machine learning model integrating carotid plaque and perivascular adipose tissue for predicting symptomatic plaques

**DOI:** 10.3389/fneur.2025.1679861

**Published:** 2025-09-24

**Authors:** Hanzhe Wang, Jingkai Xu, Chengeng Ye, Aiyun Sun, Kun Tang, Yimin Chen, Zhe Xiao, Shulan Chen, Linfeng Shao, Xiangwu Zheng, Guoquan Cao

**Affiliations:** ^1^Department of Radiology, The First Affiliated Hospital of Wenzhou Medical University, Wenzhou, China; ^2^CT Imaging Research Center, GE HealthCare China, Shanghai, China; ^3^Department of Nuclear Medicine, The First Affiliated Hospital of Wenzhou Medical University, Wenzhou, China; ^4^The First School of Medicine, School of Information and Engineering, Wenzhou Medical University, Wenzhou, China

**Keywords:** carotid plaque, atherosclerosis, perivascular adipose tissue, radiomics, Shapley Additive Explanations, stroke

## Abstract

**Rationale and objectives:**

Accurate identification of symptomatic carotid plaques remains a clinical challenge, as conventional imaging focuses mainly on luminal stenosis and lacks sensitivity to plaque vulnerability and perivascular inflammation. This study aimed to develop and validate an explainable machine learning model integrating CT-based radiomics features from carotid plaque and perivascular adipose tissue (PVAT) to identify symptomatic carotid plaques.

**Materials and methods:**

324 patients with extracranial carotid atherosclerosis and stenosis who had undergone head and neck computed tomography angiography (CTA) were retrospectively included. Three-dimensional radiomics features were extracted from segmented carotid plaque, PVAT and combined carotid plaque and PVAT (CP-PVAT) regions. Independent clinical factors were identified using univariate and multivariate logistic regression analyses. A combined model integrating the radiomics signature with selected clinical factors was developed. Models were developed and underwent internally validated using five-fold cross-validation to enhance robustness and minimize overfitting. Model interpretability was assessed using Shapley Additive Explanations (SHAP).

**Results:**

The combined model, which integrated CP-PVAT features and clinical factors, achieved excellent discriminative performance, with mean AUCs of 0.903 and 0.904 in the training and testing sets, respectively. It significantly outperformed models based solely on carotid plaque, PVAT, CP-PVAT or clinical factors (*p* < 0.05, DeLong’s test). SHAP analysis demonstrated that radiomics features provided complementary information, enhancing model interpretability and clinical relevance.

**Conclusion:**

This explainable radiomics-based model, combining CP-PVAT features with clinical risk factors, may serve as a promising tool for identifying symptomatic carotid plaques and supporting individualized cerebrovascular risk assessment.

## 1 Introduction

Atherosclerosis of the extracranial arteries, particularly the carotid arteries, plays a critical role in the pathogenesis of ischemic stroke (1, 2). While the assessment of vascular stenosis remains a cornerstone of clinical risk stratification, it is increasingly recognized that this metric alone inadequately reflects the biological complexity of plaques prone to rupture. Emerging evidence indicates that localized vascular inflammation is a key contributor to plaque formation, progression, and rupture. This inflammatory activity not only promotes plaque instability but also increases the risk of thrombosis (3).

Perivascular adipose tissue (PVAT) was initially considered a passive structure providing mechanical support to blood vessels. Over the past decades, however, its role in vascular biology has been increasingly recognized. PVAT secretes various vasoactive molecules that influence vascular tone and inflammation, and responds to signals from the adjacent vessel wall, suggesting a bidirectional and dynamic interaction between the two (4–6). In coronary artery disease, PVAT has already demonstrated significant clinical value. Changes in PVAT composition and CT attenuation have been associated with plaque inflammation, instability, and future cardiac events, making it a promising non-invasive imaging biomarker (7). Moreover, recent studies have demonstrated that PVAT in the carotid artery also plays a significant role, with changes in PVAT composition and CT attenuation found to correlate with carotid plaque vulnerability and ischemic stroke risk (8–10). However, despite these findings, the predictive value of carotid PVAT has not been fully explored, particularly regarding its spatial coupling relationship with adjacent plaques.

Computed tomography angiography (CTA) is a widely adopted imaging modality for assessing atherosclerosis due to its rapid acquisition, non-invasiveness, and broad clinical applicability (11). By providing detailed morphological information of arterial lesions, CTA offers a rich source of data for radiomic analysis. Radiomics enables the extraction of high-dimensional quantitative features—such as shape, texture, and intensity—from medical images. These features can capture subtle patterns and microstructural changes not discernible by human visual assessment, thereby supporting non-invasive risk stratification and clinical decision-making (12). While previous studies have attempted to develop predictive models for plaques or PVAT using radiomics, most of these models use two-dimensional region of interest (ROI) approaches and fail to incorporate the spatial interactions between the two, limiting the precision and clinical applicability of these models (13–16).

At the same time, the black-box nature of traditional machine learning models remains a major limitation, hindering their clinical applicability. The Shapley Additive Explanations (SHAP) method offers a solution by providing interpretable insights into feature importance and quantifying contributions to both global and instance-level predictions (17, 18).

To address these challenges, this study develops and validates a model that integrates radiomic features from carotid plaques and PVAT to enhance high-risk plaque identification and differentiate between symptomatic and asymptomatic lesions. Incorporating SHAP explanation mechanisms, the model also aims to improve interpretability and clinical utility.

## 2 Materials and methods

### 2.1 Patients

**Figure 1:**
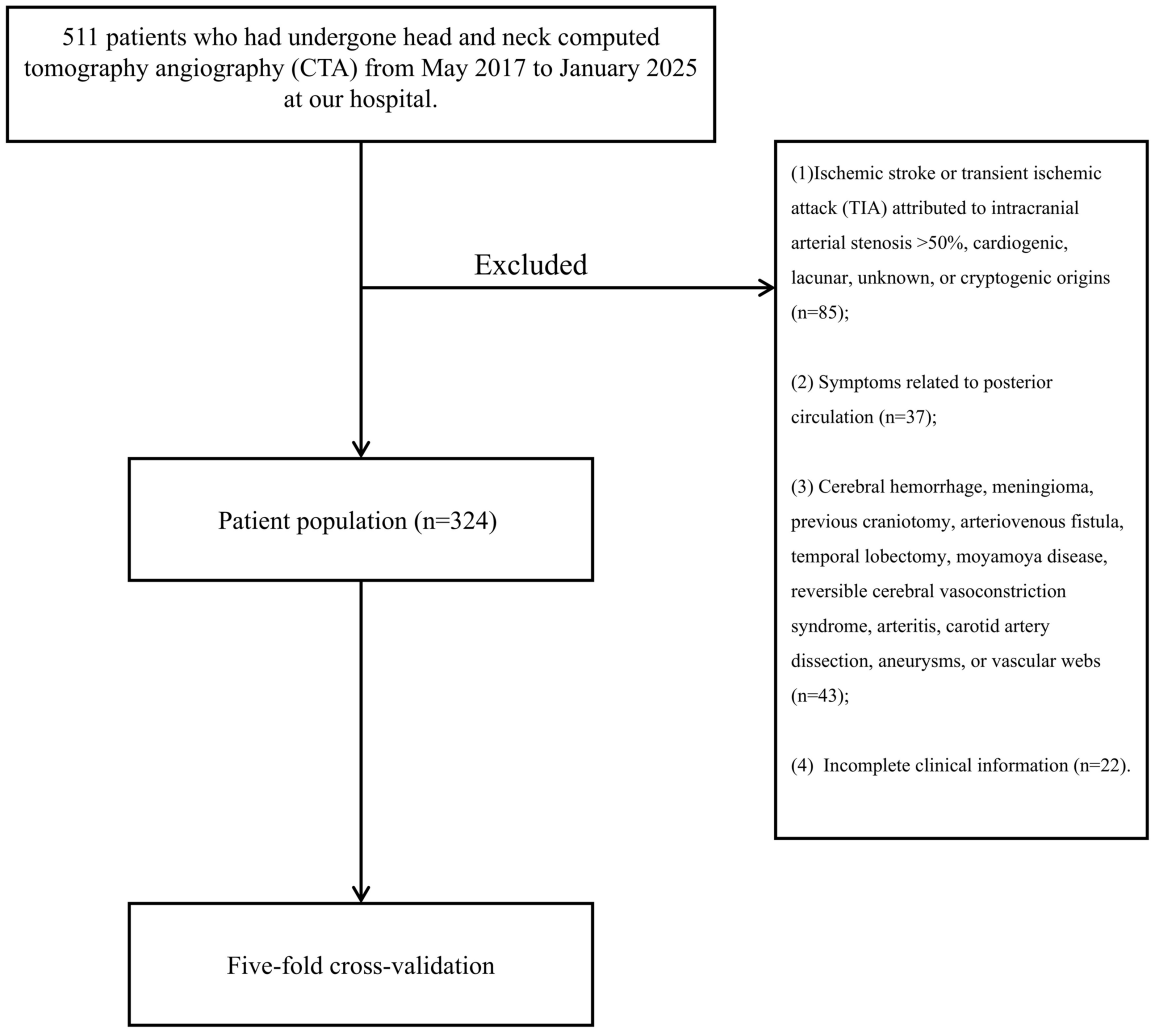
Patient selection flowchart.

Patients who had undergone head and neck CTA from May 2017 to January 2025 at our hospital were included. This study was conducted in accordance with the Declaration of Helsinki, and the protocol was approved by the Ethics Committee of the First Affiliated Hospital of Wenzhou Medical University on December 11, 2024 (KY2024-R313). The requirement for informed consent was waived by the ethics committee due to the retrospective nature of the study. Inclusion criteria were as follows: (1) a diagnosis of extracranial carotid atherosclerosis and stenosis on CTA images according to the North American Symptomatic Carotid Endarterectomy Trial criteria; (2) CTA images meeting diagnostic quality standards. The exclusion criteria: (1) ischemic stroke or transient ischemic attack (TIA) attributed to intracranial arterial stenosis >50%, cardiogenic, lacunar, or cryptogenic origins; (2) symptoms related to posterior circulation; (3) cerebral hemorrhage, meningioma, previous craniotomy, arteriovenous fistula, temporal lobectomy, moyamoya disease, reversible cerebral vasoconstriction syndrome, arteritis, carotid artery dissection, aneurysms, or vascular webs; (4) incomplete clinical data (see Figure 1).

Patients were classified into symptomatic and asymptomatic groups based on the presence of neurological symptoms within 2 weeks prior to CTA and/or radiological evidence of acute or subacute cerebral infarction identified on MRI. The symptomatic group included patients presenting with classical TIAs, ischemic events confined to the anterior circulation (i.e., the carotid artery territory), or monocular visual disturbances ipsilateral to the affected carotid plaques, such as amaurosis fugax or central retinal artery occlusion (19, 20). Classical TIA was defined as a transient episode of focal neurological dysfunction that completely resolved within 24 h, while ischemic stroke was characterized by a sudden onset of focal neurological deficits lasting more than 24 h.

### 2.2 Clinical data collection

Demographic and clinical information included sex, plaque type, age, body mass index (BMI), hyperlipidemia, history of alcohol use, smoking history, hypertension, diabetes, antihypertensive use, statin use, and antiplatelet use.

### 2.3 Segmentation on CT images

ROIs were segmented by an experienced radiologist who was blinded to patients’ clinical information. ROI delineation was performed using 3D Slicer (version 5.6.1, www.slicer.org). All CT image voxels were resampled to 1 × 1 × 1 mm to mitigate the impact of different acquisition equipment. Three ROIs were defined in this study: carotid plaque, PVAT, and the combination of carotid plaque and PVAT (CP-PVAT). Carotid plaque refers to all plaques identified in the target vessel on the axial images, with manual contouring used to accurately delineate plaque boundaries while excluding adjacent normal tissues. PVAT is defined as the adipose tissue located radially outside the vessel wall at a distance equal to the vessel diameter, with a Hounsfield unit (HU) value ranging from −190 to −30 HU (6, 21). To improve delineation accuracy, a semi-automatic delineation method based on attenuation thresholds was applied to identify PVAT. CP-PVAT refers to the combined ROI encompassing both the carotid plaque and PVAT. CTA scanning parameters are presented in Supplementary Appendix A.

### 2.4 Feature extraction and selection

Radiomic features were extracted using the PyRadiomics package integrated into 3D Slicer. Extracted features encompassed shape, first-order statistics, and texture features, including those derived from the gray level co-occurrence matrix (GLCM), gray level dependence matrix (GLDM), gray level run length matrix (GLRLM), gray level size zone matrix (GLSZM), neighborhood gray tone difference matrix (NGTDM), and wavelet-transformed images.

We evaluated both intra-rater and inter-rater reliability using the Intra-class Correlation Coefficient (ICC) on a random subset of 40 patients. For the intra-rater assessment, the primary radiologist re-segmented the plaques for these 40 cases after a two-week interval to minimize recall bias. For the inter-rater assessment, a second, independent radiologist, blinded to the initial results, segmented the same 40 cases. ICC values were calculated for all features. Only features that achieved excellent agreement (ICC > 0.8) in both the intra- and inter-rater assessments were carried forward for model development. This rigorous two-step validation ensures that our final feature set is highly robust and reproducible.

Prior to further analysis, all radiomic features were standardized using Z-score normalization. The maximal relevance minimum redundancy (mRMR) algorithm was applied to the remaining features to eliminate redundancy, followed by Least Absolute Shrinkage and Selection Operator (LASSO) regression for dimensionality reduction. Feature selection based on the mRMR and LASSO algorithms was performed using the uAI Research Portal (United Imaging Intelligence, China).

### 2.5 Development and validation of models

To minimize overfitting and fully utilize the available samples, five-fold cross-validation was conducted. This approach enabled each sample to be used for both training and testing, thereby enhancing the robustness and reliability of the model performance estimates. Among various machine learning algorithms, stochastic gradient descent (SGD) was selected to build models based on carotid plaque, PVAT, CP–PVAT, and a combined model. SHAP analysis was employed to interpret feature contributions within the models. The magnitude of each SHAP value reflects the strength of features’ impact on the prediction, while the sign indicates the direction of the effect.

**Figure 2:**
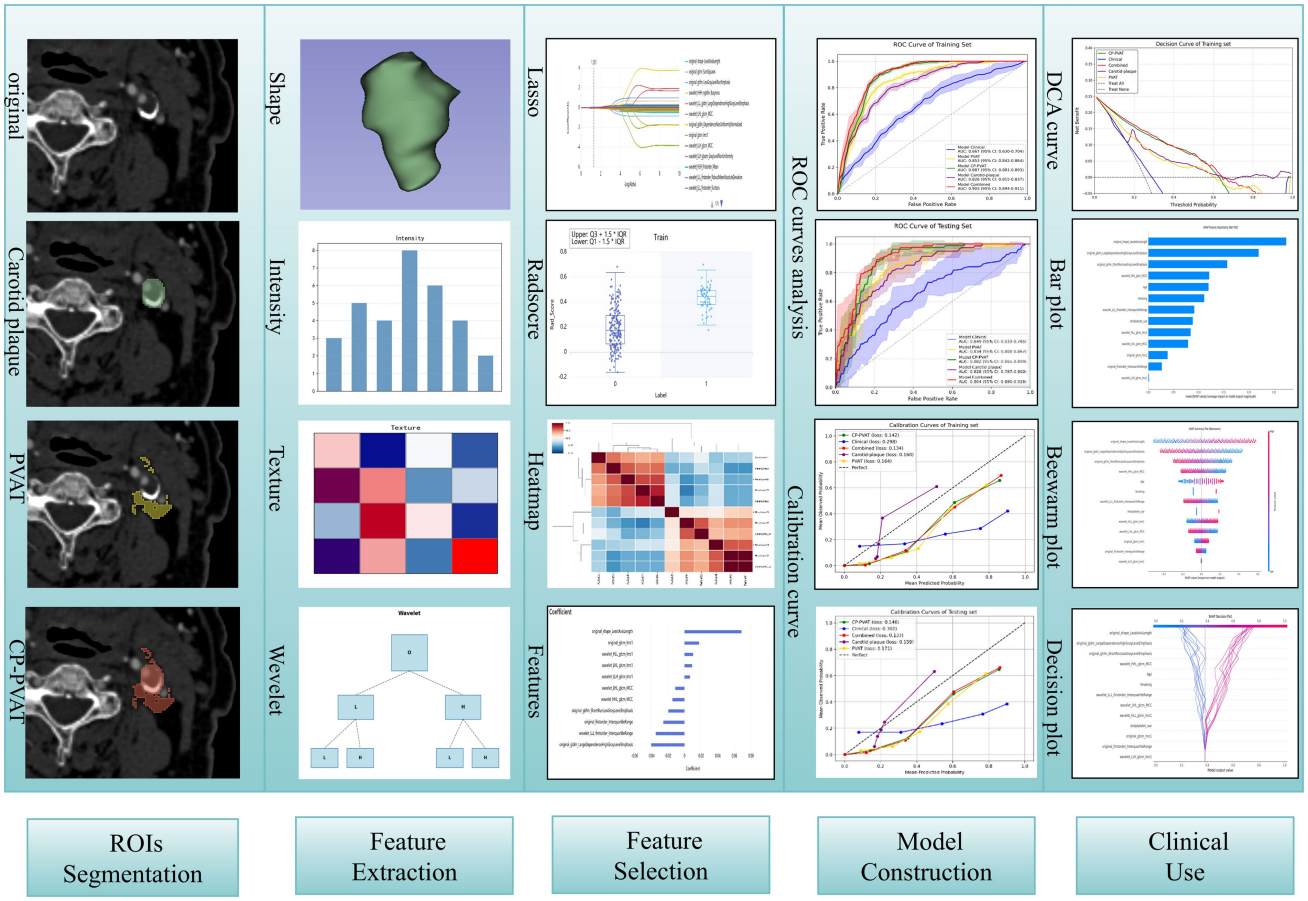
The workflow of study.

Clinical factors selection was performed using univariate and multivariate logistic regression. To prevent data leakage and ensure an unbiased performance evaluation, variable selection was strictly conducted within the training folds during the five-fold cross-validation process. In each fold, univariate logistic regression was first applied to identify clinical factors with a *p*-value < 0.05, which were then included in the multivariate regression analysis. Factors with a *p*-value < 0.05 in the multivariate analysis were retained for subsequent steps. To ensure stability, features selected in at least three of the five folds were considered for final inclusion in the model training process. Model construction based on the SGD algorithm was carried out using the uAI Research Portal (United Imaging Intelligence, China), whereas SHAP analysis was performed using Python 3. The workflow of the study is shown in Figure 2.

### 2.6 Model evaluation

Receiver operating characteristic (ROC) curves were generated for each ROI, and the area under the curve (AUC) was used to evaluate model performance. The optimal cutoff point was determined by highest Youden’s index, after which sensitivity, specificity, and accuracy were calculated. Statistical comparisons of AUCs were conducted using DeLong’s test. Calibration curves were used to assess agreement between predicted and observed symptomatic cases, while the integrated Brier score (IBS) quantified the average squared difference between predicted and observed probabilities (22). Lower IBS values indicated better model performance. Additionally, decision curve analysis (DCA) was employed to evaluate the clinical utility and net benefit of the models.

### 2.7 Statistical analysis

Statistical analyses were performed using R software (version 4.4.0, http://www.R-project.org) and IBM SPSS Statistics (version 25). A two-sided *p*-value < 0.05 was considered statistically significant. The Shapiro–Wilk test was used to assess normality. Continuous variables were compared using the Student’s t-test or the Mann–Whitney U test, as appropriate. Categorical variables were analyzed using the chi-square test or Fisher’s exact test.

## 3 Result

### 3.1 Patient characteristics

A total of 324 patients were stratified into two groups based on the presence or absence of clinical neurological manifestations: symptomatic (*n* = 82) and asymptomatic (*n* = 242). The clinical characteristics of the patients are summarized in Table 1. In the five-fold cross-validation, smoking history was found to be statistically significant in four folds in both univariate and multivariate logistic regression analyses. Age and antiplatelet use were significant in three out of the five folds (Table 2). These three factors were subsequently incorporated into the Clinical model.

**Table 1:** Clinical characteristics of patients.

Characteristic	Patients (*n* = 324)			
	Overall (*n* = 324)	Asymptomatic (*n* = 242)	Symptomatic (*n* = 82)	*p* value
Sex				0.048
Male	214 (66.05%)	152 (62.81%)	62 (75.61%)	
Female	110 (33.95%)	90 (37.19%)	20 (24.39%)	
Plaque Type				<0.001
Calcified plaque	120 (37.04%)	117 (48.35%)	3 (3.66%)	
Mixed plaque	155 (47.84%)	96 (39.67%)	59 (71.95%)	
Non-calcified plaque	49 (15.12%)	29 (11.98%)	20 (24.39%)	
Mean age (y)^a^	67.37 ± 9.01	66.67 ± 9.15	69.36 ± 8.30	0.011
BMI, kg/m^2^^a^	23.75 ± 3.05	23.75 ± 3.09	23.77 ± 2.95	0.940
Hyperlipidemia	112 (34.57%)	82 (33.88%)	30 (36.59%)	0.756
Smoking history	115 (35.49%)	73 (30.17%)	42 (51.22%)	<0.001
History of alcohol use	108 (33.33%)	76 (31.40%)	32 (39.02%)	0.259
Hypertension	187 (57.72%)	138 (57.02%)	49 (59.76%)	0.762
Diabetes	104 (32.10%)	75 (30.99%)	29 (35.37%)	0.551
Antihypertensive use	182 (56.17%)	134 (55.37%)	48(58.54%)	0.711
Statin use	113 (34.88%)	77 (31.82%)	36 (43.90%)	0.064
Antiplatelet use	91 (28.09%)	57 (23.55%)	34 (41.36%)	0.003

BMI, body mass index.

a Data are mean ± standard deviation.

**Table 2:** Univariable and multivariable logistic regression analysis of factors.

Factors	Univariable logistic regression					Multivariable logistic regression				
	*p* value					*p* value				
	Fold 1	Fold 2	Fold 3	Fold 4	Fold 5	Fold 1	Fold 2	Fold 3	Fold 4	Fold 5
Age (y)	0.010	0.030	0.060	0.080	0.010	0.010	0.020			0.010
Sex	0.140	0.030	0.080	0.020	0.100		0.480		0.130	
Smoking history	0.010	<0.001	<0.001	0.020	<0.001	0.010	<0.001	<0.001	0.150	<0.001
Hypertension	0.610	0.420	0.800	0.970	0.680					
Diabetes	0.220	0.800	0.240	0.840	0.710					
BMI	0.860	0.420	0.940	0.870	0.680					
Hyperlipidemia	0.640	0.030	0.660	0.470	0.970					
History of alcohol use	0.320	0.240	0.210	0.340	0.210					
Antihypertensive use	0.560	0.420	0.640	0.970	0.680					
Statin use	0.100	0.030	0.060	0.240	0.060		0.790			
Antiplatelet use	0.010	<0.001	<0.001	0.070	<0.001	0.020	0.070	<0.001		0.010

BMI, body mass index.

### 3.2 Feature selection and model construction

From each of the four ROIs, 851 radiomics features were extracted. Among them, 803 features from the carotid plaque, 799 from the PVAT, and 785 from the combined CP-PVAT regions demonstrated high reproducibility, with both intra- and inter-observer ICCs exceeding 0.8. Redundant and irrelevant features were eliminated using mRMR and LASSO regression. Ultimately, 8, 13, and 11 features were selected to construct the carotid plaque, PVAT, and CP-PVAT models, respectively.

### 3.3 Model performance

**Figure 3:**
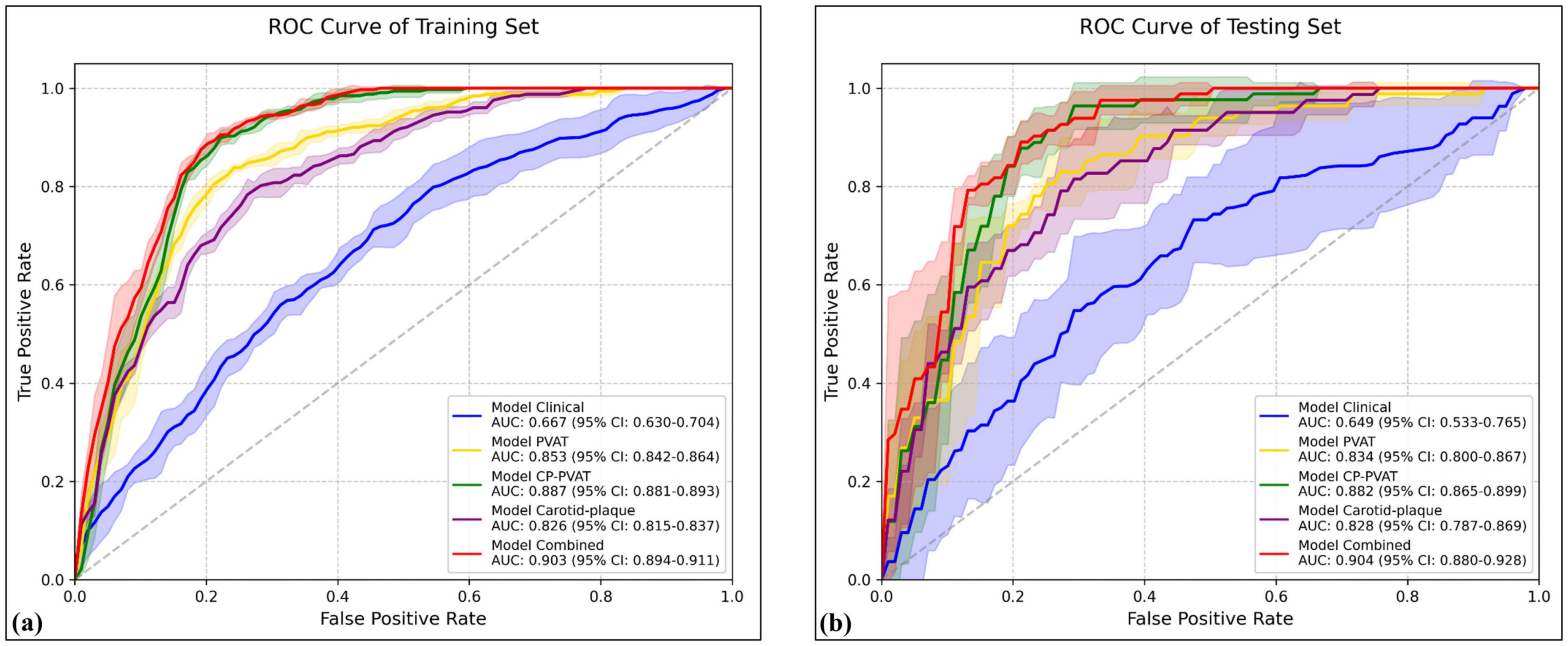
Receiver operating characteristic (ROC) curves of various models in the **(a)** training and **(b)** testing sets. Solid lines represent mean ROC curves; shaded areas represent the 95% confidence intervals.

The carotid plaque model achieved a mean AUC of 0.826 (95% CI: 0.815–0.837) in the training set and 0.828 (95% CI: 0.787–0.869) in the testing set (Figures 3a,b). The PVAT model yielded mean AUCs of 0.853 (95% CI: 0.842–0.864) and 0.834 (95% CI: 0.800–0.867) in the training and testing sets, respectively. The CP-PVAT model demonstrated the highest performance, with a mean AUC of 0.887 (95% CI: 0.881–0.893) in the training set and 0.882 (95% CI: 0.865–0.899) in the testing set (Table 3). DeLong’s test revealed the CP-PVAT model significantly outperformed both the carotid plaque model and the PVAT model (Supplementary Table S1).

**Table 3:** Prediction performance of carotid plaque, PVAT, CP-PVAT, clinical, and combined models.

Models	Training set				Testing set				Cutoff	DeLong’s test between training and testing sets (*p* value)
	Mean AUC (95% CI)	Acc	Sen	Spe	Mean AUC (95% CI)	Acc	Sen	Spe		
Carotid plaque	0.826 (0.815–0.837)	0.782	0.857	0.840	0.828 (0.787–0.869)	0.775	0.822	0.851	0.457	0.271
PVAT	0.853 (0.842–0.864)	0.796	0.793	0.798	0.834 (0.800–0.867)	0.787	0.768	0.793	0.567	0.498
CP-PVAT	0.887 (0.881–0.893)	0.819	0.863	0.804	0.882 (0.865–0.899)	0.812	0.853	0.789	0.664	0.702
Clinical	0.667 (0.630–0.704)	0.571	0.664	0.539	0.649 (0.533–0.765)	0.579	0.673	0.566	0.265	0.765
Combined	0.903 (0.894–0.911)	0.813	0.896	0.784	0.904 (0.880–0.928)	0.815	0.890	0.789	0.680	0.689

PVAT, Perivascular adipose tissue; CP-PVAT, carotid plaque and PVAT; Acc, accuracy; AUC, area under curve; CI, confidence interval; Sen, sensitivity; Spe, specificity.

The Clinical model, constructed using age, smoking history, and antiplatelet use, achieved a mean AUC of 0.667 (95% CI: 0.630–0.704) in the training set and 0.649 (95% CI: 0.533–0.765) in the testing set. The Combined model, incorporating both clinical factors and CP-PVAT radiomic features, achieving the highest performance, with a mean AUC of 0.903 (95% CI: 0.894–0.911) in the training set and 0.904 (95% CI: 0.880–0.928) in the testing set (Figures 3a,b).

DeLong’s test further confirmed that the combined model significantly outperformed all other models in both sets (all *p* < 0.05), including a notable improvement over the CP-PVAT model alone (Supplementary Table S1). Within each model group, DeLong’s test revealed no statistically significant performance difference across cross-validation folds (all *p* > 0.05), suggesting robust generalizability without overfitting (Table 3).

**Figure 4:**
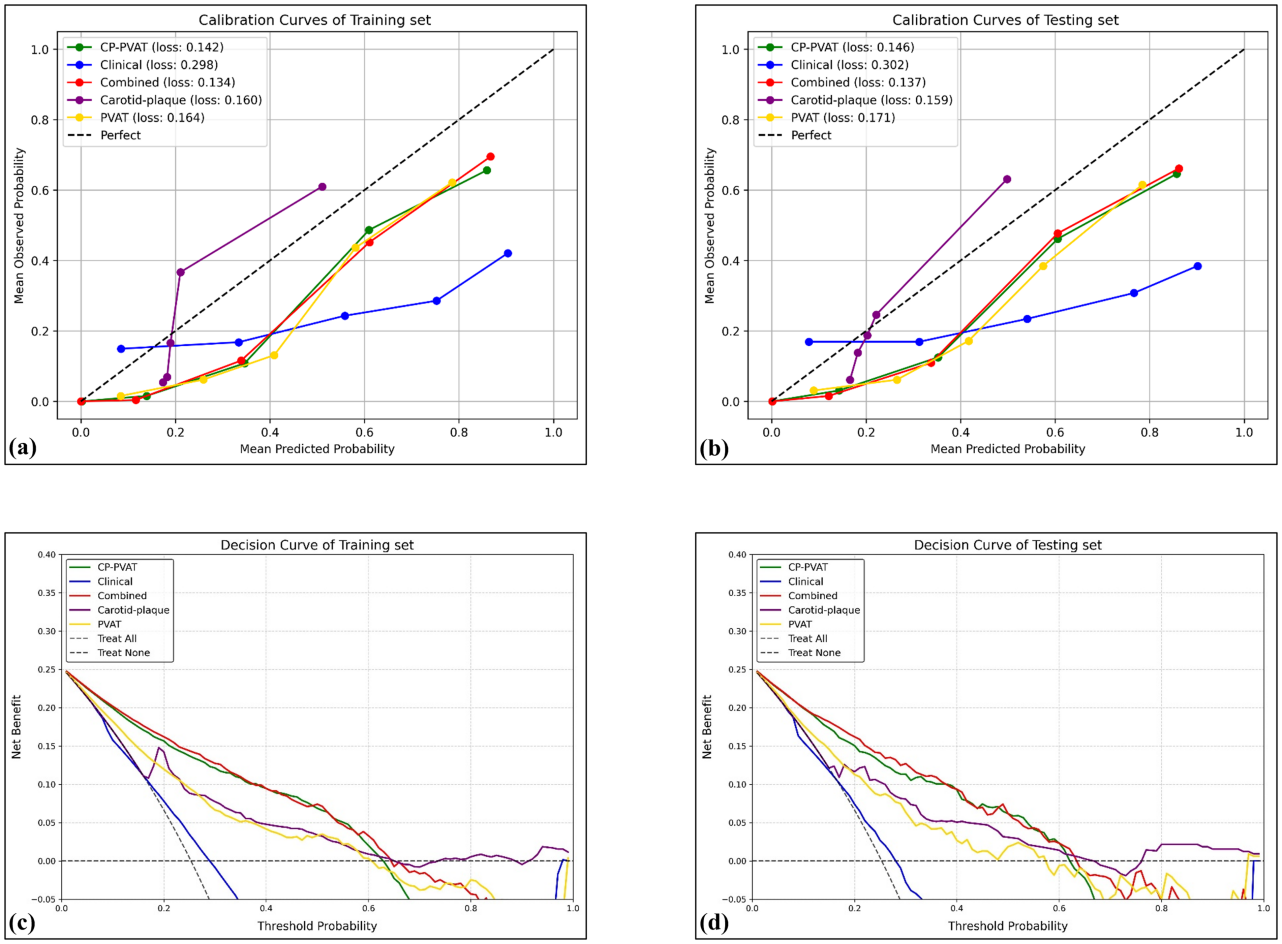
Calibration curves and DCA curves. Calibration curves revealed satisfactory calibration of the combined model with lowest Brier score in the training **(a)** and testing **(b)** sets. The *y*-axis measures the actual positive rate. The *x-*axis measures the model predicted probability. DCA of the prediction model in the training **(c)** and testing **(d)** sets showed the combined model provided a better net benefit than other radiomics models for the most of the threshold range. The y-axis measures the net benefit. The x-axis measures the threshold probability.

The Combined model’s predicted probabilities were closely aligned with observed outcomes in both the training and testing sets, as demonstrated by low IBS values (Figures 4a,b). Furthermore, DCA showed that the combined model provided the greatest net clinical benefit (Figures 4c,d).

### 3.4 Model interpretation

**Figure 5:**
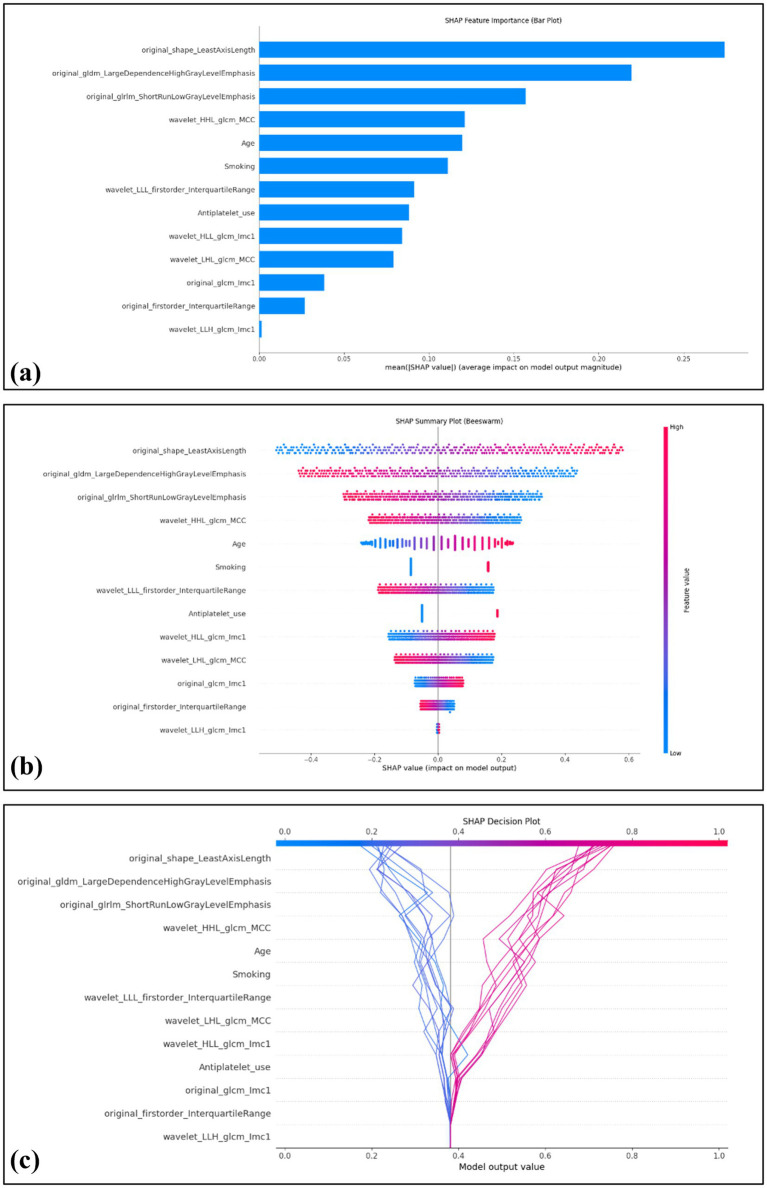
Global interpretability of the model using SHAP values. The SHAP summary bar plot depicts the overall importance of each feature in the model, ranked by mean absolute SHAP values **(a)**. The SHAP beeswarm plot displays the distribution and directional influence of individual feature values on prediction probability, with red indicating a positive effect and blue indicating a negative effect **(b)**. The SHAP decision plot visualizes 20 representative cases, including 10 correctly predicted negative cases on the left (blue lines) and 10 correctly predicted positive cases (red lines) on the right **(c)**.

The computation of SHAP values provided a transparent interpretation of the combined model’s predictions, further reinforcing its potential value in clinical decision-making. In the overall model visualization, the bar plot highlights the relative importance of each feature, with original_shape_LeastAxisLength exhibiting the highest contribution (Figure 5a). The beeswarm plot illustrates the individual impact of each feature on the predicted probability, where red and blue denote positive and negative influences, respectively (Figure 5b). Additionally, the decision plot provides a cumulative view of how each feature drives the model’s prediction across individual cases (Figure 5c).

## 4 Discussion

In this study, we developed a radiomics-based model integrating carotid plaque and PVAT features with clinical factors to identify symptomatic carotid plaques. The Combined model achieved excellent discriminative performance. Notably, ROI was jointly delineated to encompass both plaque and surrounding PVAT, enabling the extraction of spatially coupled radiomic features that reflect both structural vulnerability and inflammatory status. These results support the utility of anatomically integrated radiomics for noninvasive risk stratification in cerebrovascular disease.

Increasing evidence has highlighted the pivotal pathophysiological role of PVAT in the development and progression of atherosclerosis (23–25). Under inflammatory conditions, oxidative products and pro-inflammatory mediators released from the arterial wall can diffuse into the adjacent PVAT, inducing phenotypic alterations in adipocytes characterized by a shift toward pro-inflammatory and lipolytic profiles (23, 26). This activated state of PVAT not only contributes to vascular dysfunction and plaque instability through the paracrine secretion of inflammatory cytokines, but also exhibits a spatial gradient in adipocyte size and composition, reflecting the local intensity of vascular inflammation. Compared with stable plaques, symptomatic plaques are more frequently associated with such an inflamed and remodeled PVAT microenvironment, suggesting a close pathogenic link between PVAT activation and plaque vulnerability (27).

Carotid artery CTA enables quantitative assessment of vulnerable plaque characteristics through morphological analysis, with these pathomorphological features demonstrating significant correlations with the occurrence of cerebral ischemic events (28). Furthermore, emerging evidence suggests that radiomics-based multidimensional feature extraction from CTA imaging datasets exhibits promising potential for constructing predictive models of adverse cardiovascular outcomes, thereby offering quantifiable parameters for clinical risk stratification.

Previous studies have developed radiomics-based models to assess plaque vulnerability with varying approaches. Zhang et al. (29) proposed a CT-based radiomics nomogram to detect intraplaque hemorrhage, achieving AUCs of 0.743 and 0.811 in the training and testing cohorts, respectively. Nie et al. (16) focused solely on PVAT features to identify symptomatic plaques, reporting AUCs of 0.831 and 0.820. While these models demonstrate potential, they either lack integration of plaque morphology or provide limited insight into lesion complexity. In contrast, our Combined model integrates both clinical features and a novel CP-PVAT radiomics signature that synergistically captures both the inflammatory microenvironment (via PVAT) and intraplaque morphological heterogeneity. This approach achieved superior and more stable predictive performance. Although Chen et al. (30) attempted to integrate both plaque and PVAT features into a radiomics model (achieving AUCs of 0.883 and 0.840), their ROI was confined to the maximal cross-sectional area of the plaque. This two-dimensional segmentation may inadequately reflect the three-dimensional complexity and spatial heterogeneity inherent to atherosclerotic lesions. A key methodological strength of our study is the adoption of a 3D whole-volume segmentation approach. Although computationally simpler, the traditional 2D method—typically segmenting the plaque on its thickest cross-section—has inherent limitations: it is vulnerable to operator-dependent slice-selection bias and, more importantly, cannot capture the plaque’s full three-dimensional heterogeneity. Atherosclerotic plaques are complex structures with substantial spatial variation in volume, morphology, and composition; a single slice offers only a narrow snapshot and may miss information relevant to overall plaque burden and instability. By contrast, our 3D whole-volume analysis provides a holistic representation of the lesion, encompassing global volumetric, morphological, and textural features. This design reduces information loss and contributes to the model’s robust predictive performance, offering a superior strategy for future radiomics-based plaque risk stratification. Consistent with this rationale, our 3D whole-volume pipeline achieved superior discrimination relative to 2D slice-based analyses in both the training and testing sets, underscoring the methodological soundness of our approach.

The lack of interpretability has long been a major barrier to the clinical adoption of traditional machine learning models, significantly limiting clinicians’ understanding and trust in model predictions. In our study, SHAP was employed to interpret the model’s predictions of symptomatic carotid plaques. The SHAP bar plot indicated that original_shape_LeastAxisLength was the most influential feature in the model. This variable reflects the geometric configuration of carotid plaques, with greater values potentially representing larger plaque volumes, which are associated with hemodynamic disturbances and an increased risk of symptomatic cerebrovascular events. The SHAP beeswarm plot provided an overview of the directionality and magnitude of features’ impact on model predictions across all patients. While the SHAP decision plot demonstrated how individual features cumulatively influenced model predictions on a per-patient basis. These tools collectively provide transparent and clinically meaningful explanations for otherwise complex model outputs.

Among the clinical variables evaluated, smoking history, advanced age, and antiplatelet use were independently associated with the presence of symptomatic carotid plaques. Smoking is a well-established contributor to atherogenesis, promoting endothelial injury, oxidative stress, and vascular inflammation. These processes accelerate monocyte infiltration and foam cell formation, ultimately destabilizing atherosclerotic plaques and increasing the risk of cerebrovascular events (31–36). Advancing age similarly reflects cumulative vascular degeneration, characterized by increased arterial stiffness, impaired endothelial function, and heightened pro-inflammatory activity, all of which contribute to the development of complex and rupture-prone plaques. Interestingly, antiplatelet therapy—though intended to reduce thrombotic risk—was also significantly associated with symptomatic plaque (37, 38). This finding likely reflects a treatment-selection bias, whereby patients with more advanced carotid atherosclerosis are more frequently prescribed antiplatelet agents. Collectively, these results highlight the multifactorial nature of plaque vulnerability, where both intrinsic biological aging processes and modifiable behavioral or therapeutic factors synergistically shape clinical risk. Their inclusion in predictive modeling enhances its relevance to real-world clinical decision-making.

The clinical utility of our model is to supplement current risk assessments. Its ability to integrate radiomic features from both the carotid plaque and perivascular adipose tissue on standard CTA scans provides a non-invasive approach to potentially identify high-risk asymptomatic patients who may be missed when relying solely on luminal stenosis. Crucially, our use of SHAP analysis adds a layer of transparency by illustrating the radiomics features that drive each prediction, thereby building the confidence necessary for its potential adoption as a clinical decision-support tool.

This study has several limitations. First, it was conducted at a single center without external validation, which may limit the generalizability of the findings. Although five-fold cross-validation was employed to reduce overfitting and enhance internal robustness, external validation using multi-center cohorts is necessary to confirm the model’s performance across diverse clinical settings. Second, although ROI segmentation showed high inter- and intra-observer agreement, manual delineation remains inherently subjective—particularly in regions with ambiguous boundaries—and may compromise feature reproducibility. In addition, the model did not incorporate serological biomarkers, which may limit its biological interpretability in capturing mechanisms underlying plaque vulnerability. Future studies should consider integrating multimodal clinical and molecular data to strengthen the model’s pathophysiological relevance and prognostic utility.

## 5 Conclusion

This study presents a radiomics-based model that integrates spatially coupled features from carotid plaque and PVAT, along with clinical factors, for the identification of symptomatic carotid plaques. The Combined model showed robust performance in both training and testing sets, with added value from the joint assessment of vascular structure and perivascular inflammation. SHAP-based interpretability enhanced transparency and clinical insight. These findings indicate that anatomically informed radiomics models hold promise for noninvasive cerebrovascular risk stratification.

## Data Availability

The raw data supporting the conclusions of this article will be made available by the authors, without undue reservation.

## Glossary

PVAT: Perivascular adipose tissue
CTA: Computed tomography angiography
ROI: Region of interest
SHAP: Shapley Additive Explanations
TIA: Transient ischemic attack
BMI: Body mass index
CP-PVAT: Carotid plaque and PVAT
HU: Hounsfield unit
GLCM: Gray level co-occurrence matrix
GLDM: Gray level dependence matrix
GLRLM: Gray level run length matrix
GLSZM: Gray level size zone matrix
NGTDM: Neighborhood gray tone difference matrix
ICC: Intraclass correlation coefficient
mRMR: Maximal relevance minimum redundancy
LASSO: Least Absolute Shrinkage and Selection Operator
SGD: Stochastic gradient descent
ROC: Receiver operating characteristic
AUC: Area under the curve
IBS: Integrated Brier score
DCA: Decision curve analysis
