# Supplementary material for: An explainable CT-based machine learning model integrating carotid plaque and perivascular adipose tissue for predicting symptomatic plaques
Source: Front Neurol. 2025 Sep 24;16:1679861. doi: 10.3389/fneur.2025.1679861 (PMC12504078; doi:10.3389/fneur.2025.1679861)
Supplement: Supplementary file 1 [file Table_1.docx]

**Supplemental Materials**

**Supplemental Appendix A:** CTA scanning parameters.

The carotid CTA were carried out in helical mode with parameters as follows: tube voltage of 120 kVp, pitch of 1.0, slice interval and thickness of 0.625 mm, rotation time of 0.28 s, and scanning ranged from the aortic arch to the cranial vertex. Nonionic-iodinated contrast agent (350 mgI/mL, Omnipaque 350, GE Healthcare, Shanghai, China) was injected intravenously into the right cubital vein at a rate of 4.0 mL/s using an automatic injector with a bolus of 40 mL and followed by 30-mL saline flush at the same injection rate. The bolus-tracking technology was adopted with trigger point at the ascending aorta, trigger threshold of 120 HU and a delay time of 5 s.

**Supplemental Table S1:** Delong’s test of models

| Model | Set | *p* value |
| --- | --- | --- |
| CP-PVAT vs carotid plaque | Training set | <0.001 |
|  | Testing set | 0.001 |
| CP-PVAT vs PVAT | Training set | 0.003 |
|  | Testing set | 0.078 |
| CP-PVAT vs Clinical | Training set | <0.001 |
|  | Testing set | <0.001 |
| Combined vs carotid plaque | Training set | <0.001 |
|  | Testing set | <0.001 |
| Combined vs PVAT | Training set | <0.001 |
|  | Testing set | 0.020 |
| Combined vs Clinical | Training set | <0.001 |
|  | Testing set | <0.001 |
| Combined vs CP-PVAT | Training set | <0.001 |
|  | Testing set | 0.023 |
